# The clinical characteristics analysis of serum markers for the cardiovascular system in early-stage COVID-19 patients

**DOI:** 10.3389/fcvm.2024.1401586

**Published:** 2024-07-26

**Authors:** Xi Cao, Yong-Li Xie, Jian-ying Yi, Zhi-li Liu, Dong-dong Zhang, Ying-ying Yue, Tian-ning Li, Chun-lei Zhou, Hong Mu

**Affiliations:** ^1^ Department of Clinical Laboratory, Tianjin First Central Hospital, Tianjin, China; ^2^ Department of Clinical Laboratory, Tianjin Stomatological Hospital, School of Medicine, Nankai University, Tianjin, China; ^3^ Tianjin Key Laboratory of Oral and Maxillofacial Function Reconstruction, Tianjin Stomatological Hospital, Tianjin, China; ^4^ Department of Clinical Laboratory, The Third Central Hospital, Tianjin, China

**Keywords:** omicron, cardiovascular system injury, B-type natriuretic peptide, D-dimer, procalcitonin

## Abstract

**Background:**

This study aimed to investigate alterations in serum markers [creatine kinase-MB (CKMB), cardiac troponin T (cTnT), myoglobin (Myo), B-type natriuretic peptide (BNP), D-dimer (DD), procalcitonin (PCT) and interleukin-6 (IL6)] in early Omicron variant infection and analyzed their correlation with clinical parameters.

**Methods:**

Retrospective analysis of 1,138 mild/asymptomatic cases at Tianjin First Central Hospital, including age, gender, serum markers and nucleic acid test results. Statistical analysis used SPSS software, version 24.0.

**Results:**

Elevated cTnT, BNP (125–400), and DD (0.55–1.10) levels were prevalent at 12.92%, 15.64%, and 14.50%, respectively. Females had significantly higher proportions with slightly elevated BNP (19.34%) and DD (19.69%) levels. Patients over 35 had a higher proportion of slight elevation in BNP (20.00%). Abnormal levels of serum markers were significantly associated with older age, increased PCT and IL6 levels, as well as delayed nucleic acid clearance. Additionally, levels of immunoglobulin G (IgG) were notably reduced in these cases. Patients with prolonged nucleic acid clearance (>14 days) had higher BNP and DD levels upon admission. Logistic regression identified PCT (OR = 237.95) as the most significant risk factor for abnormal serum markers for cardiovascular system injury.

**Conclusion:**

Early Omicron infection might do subclinical damage to the cardiovascular system. Elevated cTnT, BNP and DD levels were correlated with age, gender, inflammatory factors, and IgG. Notably, high PCT level emerged as the most robust predictor of abnormal serum biomarkers.

## Introduction

The COVID-19 pandemic had emerged as a global health crisis of substantial magnitude. While severe respiratory failure was the primary cause of mortality in patients with coronavirus disease 2019 (COVID-19) (1), a considerable number of patients had also suffered from cardiovascular diseases (2, 3). These included not only cardiac injury but also thromboembolic events (4, 5). Previous research indicated that individuals with severe symptom, pre-existing medical conditions or advanced age were at a higher risk of developing COVID-19-related cardiovascular diseases (6).

Indeed, the impact of COVID-19 extends beyond severe cases, as the cardiovascular health of individuals with mild cases may also be compromised. A previous report indicated that cardiac involvement was present even in COVID-19 patients who exhibited asymptomatic or mild symptom (7). Rajpal and colleagues (8) demonstrated that some young patients continued to experience myocardial inflammation following recovery from COVID-19, despite having initially been asymptomatic or experiencing only mild symptoms. With the emergence of the Omicron variant as the dominant strain in circulation, those infected with the Omicron variant tend to be younger, had fewer comorbidities, and experienced lower disease severity and mortality, with asymptomatic or mild symptom being more prevalent (9–11). This had raised concerns about the potential for cardiovascular system injury in Omicron-infected individuals, particularly those who were asymptomatic or had only mild symptoms. To date, most studies on cardiovascular serum markers associated with Omicron infection have primarily focused on severe cases or elderly patients with chronic diseases. The status of the cardiovascular system in asymptomatic or mild cases remains unknown, and it is still unclear whether these individuals are at risk for cardiovascular complications.

Serum markers such as CK-MB, cTnT, Myo, BNP, and DD offered considerable clinical utility for rapid and precise assessment of the patient's cardiovascular status. In order to fill this gap in relevant knowledge, we conducted a retrospective study that evaluated serum markers suggestive of cardiovascular system injury (CK-MB, cTnT, Myo, BNP, and DD) adjusted for demographic characteristics and other relevant markers, in a cohort of 1,138 asymptomatic or mild patients with Omicron infection.

## Material and methods

### Participants and data source

The study was conducted at Tianjin First Central Hospital in Tianjin, China, spanning from March 2022 to June 2023. The COVID-19 strain from these infected patients had been sequenced and identified as the Omicron variant. Inclusion criteria comprised: (1) confirmation of asymptomatic or mild COVID-19 cases, (2) performance of nasopharyngeal swab tests more than twice during the hospital stay with an interval time >24 h, and (3) availability of results for CKMB, cTnT, Myo, BNP, and DD upon admission. Exclusion criteria included: (1) patients with liver and/or kidney dysfunction, (2) patients with serious inflammatory diseases such as chronic pulmonary disease, digestive system disease, immunological diseases, and others, (3) patients with cerebral infarction or cardiovascular issues at admission, or (4) patients with missing data. A total of 1,138 COVID-19 patients were included, comprising 589 asymptomatic and 549 mild cases. Data collection involved recording symptoms at admission, laboratory results, and nucleic acid test outcomes from the COVID-19 rehabilitation ward at Tianjin First Central Hospital. The diagnosis of asymptomatic or mild cases followed guidelines provided by the National Health Commission of China. Mild patients primarily exhibited clinical symptoms such as fever, slight fatigue, and disorders of smell and taste. In CT scans of the lungs, there were no characteristic interstitial changes typically seen in COVID-19 infections. The diagnosis and grouping of patients depended on clinical infectious disease doctors.

Chest CT scans were reviewed by specialized physicians. None patients showed radiological signs of pneumonia. We had reviewed the electronic medical records of all enrolled patients and confirmed that upon admission, none had history of long-term medication use and exhibited clinical symptoms indicative of cardiovascular disease. Those were classified into the abnormal group if they exhibited values for one or more serum markers suggestive of cardiovascular system injury (CK-MB, cTnT, Myo, BNP, and DD) that exceeded the normal reference ranges. Patients were categorized into two groups based on the presence of abnormal serum markers for cardiovascular system injury: those with normal indicators (652 patients) and those with elevated indicators (486 patients). Reference values for serum markers suggestive of cardiovascular system injury (CKMB, cTnT, Myo, BNP, and DD) were established as follows: CKMB: 0.30–3.61 ng/ml, Myo: 25–58 ng/ml, cTnT: 3–14 ng/L, BNP: 5–125 pg/ml, DD: 0.03–0.55 mg/L fibrinogen equivalent unit (FEU). In this study, patients with CKMB (>3.61 ng/ml), cTnT (>14 ng/L), or Myo (>58 ng/ml) were classified as having abnormal elevation. The normal range for BNP is 0–125, with levels between 125 and 400 indicating a potential risk of heart failure, and levels exceeding 400 suggesting a significantly increased incidence of heart failure (12). The normal range for D-Dimer is 0–0.55. Levels exceeding twice the critical value indicated an increased risk of intravascular coagulation (13). Therefore, abnormal elevation of BNP and DD was categorized into two levels (BNP: 125–400 and >400; DD: 0.55–1.10 and >1.10). In order to explore the influence of other factors, patients were categorized according to their clinical symptoms, gender, and age. Within the patient cohort, there were 589 asymptomatic patients and 549 patients. The gender distribution was nearly equal, with 564 male and 574 female patients. To ensure roughly equal numbers of patients in both groups, we selected the median age of the enrolled patients as the threshold: 35 years (Year ≤35: *n* = 593; Year >35: *n* = 545).

Serum IgG/IgM against the SARS-CoV-2 protein were assessed using magnetic particle chemiluminescence (Biology and Science, China). CKMB, cTnT, Myo, BNP, DD, PCT, and IL6 were evaluated through quantitative electrochemiluminescence immunoassay (Ren mai, China). Nasopharyngeal swabs were collected daily from all patients to minimize the occurrence of false-negative results in hospital. Each patient's sample was tested using commercial SARS-CoV-2 kits from two different manufacturers (Sheng Xiang/Bo Jie, China). According to the ninth edition of the China Novel Coronavirus Pneumonia Prevention and Control Protocol, COVID-19 patients were advised to be isolated for at least 14 days. So we set limit at 14 days, the patients were then divided into two groups: one group included patients whose nucleic acid turned negative ≤14 days (*n* = 459), and the other group included patients whose nucleic acid turned negative >14 days (*n* = 679).

RT-PCR assays targeting two genes: the open reading frame of 1ab (ORF1ab) and the nucleocapsid protein (N). SARS-CoV-2 RNA detection were performed, and the cycle threshold value (Ct-value) less than 40 with an S- shape amplification curve was defined as positive. The clinical classification for COVID-19 patients were based on the China Technical Guidelines for Laboratory Testing for COVID-19. The date of diagnosis was defined as the day when the first sample tested positive for SARS-CoV-2 by RT-PCR. Discharge criteria adhered to WHO guidance, requiring two consecutive negative PCR swabs taken more than 24 h apart, with no clinical symptoms. This study received approval from the Medical Ethics Committee of Tianjin First Central Hospital (Ethics Committee archiving No. 2022N052KY) and adhered to the principles outlined in the Declaration of Helsinki.

### Statistical analysis

Statistical analysis was conducted using SPSS 24.0 for Windows software (SPSS, Chicago, IL). Normally distributed data were presented as the mean ± standard deviation. Between-group comparisons of variables with a normal distribution and homogeneous variances were performed using the *t*-test. Non-normally distributed data were expressed as the median (upper quartile and lower quartile), and the non-parametric Mann–Whitney *U*-test was used for between-group comparisons of non-normally distributed or heterogeneously variant data. Categorical characteristics were described in counts and percentages (%), with the chi-squared test used for categorical variables. Risk factors were analyzed through univariate logistic regression, providing the *P*-value and odds ratio (OR), accompanied by its 95% confidence interval (CI). Significance was determined at *P* < 0.05, and a 95% CI for OR; an OR >1 indicated an increased risk of the event, while an OR <1 indicated a decreased risk. A *P*-value of <0.05 was considered statistically significant, and *P* < 0.001 was considered highly statistically significant. The covariates included in the models were selected through a stepwise regression analysis. All covariates that demonstrated a statistically significant impact on the dependent variable were retained in models (*P* < 0.05).

## Results

In this cross-sectional retrospective study, a total of 1,138 COVID-19 patients were included, consisting of 589 asymptomatic and 549 mild cases. The male population constituted approximately 50.42% of the asymptomatic group and 48.63% of the mild group, with mean ages of 32.46 ± 0.61 and 33.94 ± 0.61 years old, respectively (Table 1). Clinical characteristics of biochemistry and hematology were summarized in Table 1, revealing a significant difference only in IgG levels between asymptomatic and mild patients, with mild patients exhibiting higher levels (19.27 ± 1.73 vs. 26.16 ± 2.15) (*P* < 0.05). Among the total COVID-19 patients, a relatively high proportion had elevated levels of cTnT, BNP, and DD (12.92%, 17.66%, and 19.77%, respectively) (Table 2). In contrast, the proportions of patients with elevated levels of CKMB and Myo were lower, at 1.83% and 2.99%, respectively (Table 2).

**Table 1:** Laboratory characteristics of 1,138 asymptomatic or mild patients (mean ± SD).

Variables	Asymptomatic patients	Mild patients	*P*-value
Cases, *n*	589	549	–
Male, *n* (%)	297 (50.42%)	267 (48.63%)	0.546
Age (years)	32.46 ± 0.61	33.94 ± 0.61	0.091
CKMB (ng/ml)	1.12 ± 0.04	1.06 ± 0.04	0.349
cTnT (ng/L)	9.40 ± 0.35	9.24 ± 0.31	0.728
Myo (ng/ml)	27.27 ± 0.79	28.12 ± 1.39	0.596
BNP (pg/ml)	80.68 ± 4.07	81.50 ± 5.28	0.910
DD (mg/L FEU)	0.41 ± 0.03	0.40 ± 0.02	0.756
IgG (g/L)	19.27 ± 1.73	26.16 ± 2.15	**0** **.** **011**
IgM (g/L)	0.54 ± 0.16	0.42 ± 0.07	0.486
PCT (ng/ml)	0.06 ± 0.005	0.058 ± 0.003	0.270
IL6 (pg/ml)	12.27 ± 0.36	11.78 ± 0.37	0.337

Reference values: CKMB: 0.30–3.61 ng/ml, Myo: 25–58 ng/ml, cTnT: 3–14 ng/L, BNP: 5–125 pg/ml, DD: 0.03–0.55 mg/L FEU, PCT: 0–0.046 ng/ml, IL6: 0–7 pg/ml.

Bold values statistically significant differences (*P* < 0.05).

**Table 2:** Proportion of subjects with elevated biomarkers for cardiac injury or thromboembolism by degree of disease severity, sex, and age.

Variables	Asymptomatic (*n* = 589)	Mild (*n* = 549)	Male (*n* = 564)	Female (*n* = 574)	Year ≤35 (*n* = 593)	Year >35 (*n* = 545)	Total (*n* = 1,138)
CKMB(>3.61 ng/ml)	2.21% (13)	1.09% (6)	1.95% (11)	1.39% (8)	1.52% (9)	1.83% (10)	1.83% (19)
cTnT (>14 ng/L)	12.73% (75)	13.11% (72)	14.01% (79)	11.85% (68)	11.47% (68)	14.50% (79)	12.92% (147)
Myo (>58 ng/ml)	3.23% (19)	2.73% (15)	4.96% (28)	1.05% (6)***	2.36% (14)	3.67% (20)	2.99% (34)
BNP (125–400 pg/ml)	16.64% (98)	14.57% (80)	11.88% (67)	19.34% (111)**	11.64% (69)	20.00% (109)^###^	15.64% (178)
BNP (>400 pg/ml)	1.70% (10)	2.37% (13)	1.77% (10)	2.26% (13)	1.85% (11)	2.20% (12)	2.02% (23)
DD (0.55–1.10 mg/L FEU)	15.11% (89)	12.90% (76)	9.22% (52)	19.69% (113)***	15.18% (90)	13.76% (75)	14.50% (165)
DD (>1.10 mg/L FEU)	5.60% (33)	4.92% (27)	4.26% (24)	6.27% (36)	5.56% (33)	4.95% (27)	5.27% (60)

Percentage values represent the percent of individuals with elevated levels of the respective biomarker (the absolute number of individuals).

Compare between different genders: **P-values <0.01; ***P-values <0.001.

Compare between different ages: ^###^*P*-values <0.001.

A comparative analysis was conducted, examining symptom presentation, gender, and age (older than 35). The results indicated a significantly higher proportion of male patients with elevated Myo levels (4.96%) compared to female patients (1.05%) (Table 2). However, male patients exhibited significantly lower proportions of slight elevated BNP (125–400) (11.88%) and DD (0.55–1.10) (9.22%) compared to female patients (19.34% and 19.69%, respectively) (Table 2). Notably, among patients older than 35, the proportion with slight elevated BNP (125–400) (20.00%) was significantly higher than in those younger than 35 (11.64%) (Table 2). Surprisingly, no significant association was found between the proportion of serum markers for cardiovascular system injury and the presence or absence of symptoms in patients categorized as asymptomatic and mild.

The data indicated that patients with elevated serum markers had a higher proportion of females, higher age, elevated IL6 and PCT levels, and lower IgG levels (*P* < 0.05) (Table 3). Additionally, the abnormal group exhibited a relatively prolonged nucleic acid clearance time compared to normal patients. Further stratification based on the days for nucleic acid clearance resulted in two groups: those with negative nucleic acid within 14 days (459 patients) and those with negative nucleic acid after 14 days (679 patients). Patients with nucleic acid turning negative after 14 days displayed higher age, increased IL6 levels, elevated BNP and DD levels, and lower IgG levels (*P* < 0.05) (Table 4).

**Table 3:** Comparison of 652 COVID-19 patients vs. 486 COVID-19 patients with abnormal cardiovascular markers.

Variables	Normal group	Abnormal group	*P*-value
Cases, *n*	652	486	–
Male, *n* (%)	349 (53.53%)	215 (44.24%)	**0** **.** **002**
Mild, *n* (%)	319 (48.93%)	230 (47.33%)	0.593
Age (years)	32.22 ± 0.51	34.44 ± 0.76	**0** **.** **011**
IgG (g/L)	29.03 ± 2.08	14.04 ± 1.53	**<0** **.** **001**
IgM (g/L)	0.59 ± 0.15	0.34 ± 0.04	0.153
PCT (ng/ml)	0.051 ± 0.002	0.075 ± 0.005	**<0** **.** **001**
IL6 (pg/ml)	10.92 ± 0.29	13.51 ± 0.46	**<0** **.** **001**
Days for nucleic acid clearance	15.01 ± 0.20	16.61 ± 0.23	**0** **.** **003**

Bold values statistically significant differences (*P* < 0.05).

**Table 4:** Characteristics differences of 1,138 COVID-19 patients between different groups dividing by the time of viral clearance.

Variables	Nucleic acid turned negative ≤14 days	Nucleic acid turned negative >14 days	*P*-value
Cases, *n*	459	679	–
Male, *n* (%)	239 (52.07%)	325 (47.86%)	0.164
Mild, *n* (%)	236 (51.42%)	313 (46.10%)	0.078
Age (years)	31.51 ± 0.65	34.30 ± 0.58	**0** **.** **002**
CKMB (ng/ml)	1.13 ± 0.045	1.07 ± 0.033	0.261
cTnT (ng/L)	9.30 ± 0.39	9.34 ± 0.29	0.932
Myo (ng/ml)	27.41 ± 0.95	27.86 ± 1.14	0.782
BNP (pg/ml)	69.94 ± 4.73	88.61 ± 4.50	**0** **.** **006**
DD (mg/L FEU)	0.35 ± 0.016	0.44 ± 0.031	**0** **.** **015**
IgG (g/L)	38.50 ± 3.00	11.82 ± 0.88	**<0** **.** **001**
IgM (g/L)	0.67 ± 0.21	0.35 ± 0.04	0.070
PCT (ng/ml)	0.059 ± 0.004	0.063 ± 0.004	0.528
IL6 (pg/ml)	10.73 ± 0.33	12.91 ± 0.37	**<0** **.** **001**

Bold values statistically significant differences (*P* < 0.05).

Subsequently, logistic regression analysis was performed to evaluate the risk associated with significant parameters in predicting abnormal elevation in serum markers for cardiovascular system injury. The study focused on identifying the primary factors influencing the occurrence of abnormal elevation. The results revealed that the OR (95% CI) value of IgG was 0.99 (0.98–1.00) (*P* < 0.001). Additionally, increasing age, female gender, and higher levels of IL6 and PCT were identified as significant risk factors, with ORs (95% CIs) of 1.02 (1.01–1.03), 1.82 (1.40–2.37), 1.03 (1.01–1.04), and 237.95 (18.66–3,034.98), respectively, all achieving statistical significance at *P* < 0.001 (Table 5). Notably, PCT exhibited the strongest association (OR = 237.95) and emerged as the most impactful single risk factor for predicting abnormal elevation in serum markers for cardiovascular system injury (Table 5).We further made multiple regression analysis to test the combined contribution of several factors, and found the model including age, gender and PCT appeared to be the most combined risk factor for predicting abnormal elevation in serum markers for cardiovascular system injury (OR (95% CI) = 260.59 (34.23–370.34), *P* < 0.001).

**Table 5:** Association of abnormal cardiovascular markers with demographics and inflammatory factors.

Variables	Regression coefficient	Wald value	OR (95% CI)	*P*-value
Gender (female)	0.60	19.79	1.82 (1.40–2.37)	**<0** **.** **001**
Age	0.02	15.39	1.02 (1.01–1.03)	**<0** **.** **001**
IgG	−0.01	15.18	0.99 (0.98–1.00)	**<0** **.** **001**
PCT	6.50	18.40	237.95 (18.66–3,034.98)	**<0** **.** **001**
IL6	0.03	11.62	1.03 (1.01–1.04)	**0** **.** **001**
Gender + Age	4.17	16.15	64.86 (8.48–496.27)	**<0** **.** **001**
Gender + Age + PCT	4.72	60.46	260.59 (34.23–370.34)	**<0** **.** **001**
Gender + Age + PCT + IL6	4.60	72.66	125.25 (33.48–285.67)	**<0** **.** **001**
Gender + Age + IgG + PCT + IL6	4.84	87.37	124.01 (45.92–349.90)	**<0** **.** **001**

Bold values statistically significant differences (*P* < 0.05).

## Discussion

In the current study, we found that during the early stages of Omicron infection, a small percentage of individuals with asymptomatic or mild COVID-19 by the Omicron variant have elevated biomarkers for cardiac and thromboembolic disease. These biomarkers demonstrated a positive correlation with age, female gender, elevated inflammatory factors (PCT and IL-6), and a negative correlation with IgG antibody titers. Notably, high levels of PCT emerged as the most prominent risk factor for the abnormal elevation of serum markers suggestive of cardiovascular system injury. Our data implied that even among asymptomatic or mild Omicron-infected individuals, there may exist a potential risk of subclinical injury in cardiovascular system.

In our results, elevated serum markers (cTnT, BNP, and DD) demonstrated a positive correlation with age, female gender, and levels of inflammatory factors (PCT and IL6), and a negative correlation with IgG antibody levels. Cardiac markers (CKMB, cTnT, and Myo) are crucial in diagnosing and risk stratifying acute myocardial infarction, it was noteworthy that approximately 2% of patients exhibited elevated levels of CKMB and Myo. This finding suggested that myocardial tissue inflammation may occur in asymptomatic or mild cases, albeit relatively infrequently. It led to speculation that, during the initial invasion of the COVID-19 virus into the human body, it targetted myocardial cells, resulting in an increase in myocardial enzyme levels. Compared to cTnT, CKMB and Myo rise earlier in myocardial injury but had a shorter half-life and lower specificity. The levels of CK-MB and Myo might had already returned to normal by the time patients were admitted for testing. Notably, a relatively high proportion of patients with elevated cTnT levels was observed, accounting for 12.92%, which was close to 12% of COVID-19 patients in Wuhan experienced acute heart injuries (14). Among these markers, cTnT was the most specific marker that appears after acute myocardial inflammation or infarction (15). Our results indicated that the Omicron strain did cause subclinical injury to the heart, and even during the early stage of Omicron infection, the viral impact on myocardial tissue was not negligible. Additionally, other studies found that after COVID-19 infection, the levels of hs-TnI, sST-2 and VCAM-1 were closely correlated with major cardiovascular event and mortality (16, 17), which further emphasized clinical significance of abnormally elevated troponin in patients with primary infection.

BNP levels were independent of other clinical factors, rendering it a valuable indicator of cardiac risk, particularly for heart failure (18). DD, a fibrin degradation product resulting from thrombus fibrinolysis, had previously been associated with an increased risk of venous thromboembolism (VTE). Previous studies indicated that cTnT, BNP, and DD levels rise as a late manifestation in severe COVID-19, with markedly elevated levels linked to significant in-hospital mortality (19–21). In this study, the majority patients showed slight elevations in BNP and DD. Additionally, a small percentage of patients with BNP greater than 400 or DD levels exceeding 1.10 are identified as “high risk of heart failure, it still need sufficient clinical attention (12, 13)”.

In terms of gender differences, we noted a significantly higher proportion of male patients with elevated Myo levels compared to female patients. This discrepancy may be attributed to higher androgen levels and increased muscle mass in men, resulting in elevated myoglobin levels (22). Contrary to previous reports (23), our findings regarding BNP and DD differed. Cheng et al. reported higher levels of DD and BNP in men with mild or critically ill COVID-19, while our study indicated significantly lower proportions in male patients. These variations might be linked to the severity of COVID-19 infection and the prevalence of comorbidities, which were more common in males. Excluding comorbidities, our study suggested that BNP and DD were more likely to exhibit slight increases in females in the early stages of Omicron infection. We speculated that higher estrogen levels in women enhanced ACE2 activity and expression, potentially leading to a mild reactive increase in BNP and DD shortly after Omicron infection (24).

A previous study demonstrated that men aged 54 or older with SARS-CoV-2 infection and mild to moderate COVID-19 exhibited significantly increased troponin levels early after infection (25). Our study results showed that patients older than 35 years had a higher proportion of abnormally elevated BNP, with no significant difference in abnormal cTnT proportions across different age groups. However, there was no significant association between the proportion of abnormal increased serum markers for the cardiovascular system injury and the presentation of patient symptoms (asymptomatic or mild). This suggested that the degree of abnormal increased serum markers for the cardiovascular system caused by the virus might not be significantly linked to cardiovascular symptoms. Reports of sudden cardiac death following uneventful COVID-19 raise concerns about potential enduring myocardial damage (26). This was supported by the detection of persistent SARS-CoV-2 RNA in various organs, including the heart (27, 28). Our results contributed additional insights by highlighting gender and age differences. These differences suggested that there may exist subclinical cardiovascular system injury, degree of elevation of clinical serum markers varied depending on gender and age. Furthermore the influence in COVID-19 infection might extend to non-cardiac entities such as pulmonary hypertension due to acute pulmonary embolism (29).

In cases of moderate or severe COVID-19, cardiac injury and thromboembolic events were common occurrence, which was likely a result of a combination of cytokine release, inflammation, and viral damage (30). Among the five indicators examined in our study, CKMB, Myo and cTnT are directly associated with myocardial damage, BNP is linked to heart failure, and DD is correlated with cardiovascular thrombotic events. Our results revealed that patients with abnormal combination of indicators (CKMB, cTnT, Myo, BNP, and DD) exhibited significantly lower levels of IgG and higher levels of inflammatory factors (IL6 and PCT). The positive association between cardiac biomarkers and PCT and IL-6 represents tissue damage caused by an excessive inflammatory reaction (31). Contrastingly, IgG antibodies might provide protection to organs by neutralizing a portion of the virus, binding to the viral S-protein, and interfering with its interaction with the ACE2 receptor (32). Our findings aligned with this, indicating that normal patients exhibit higher serum IgG levels.

Previous studies had linked PCT, the precursor of the hormone calcitonin, with the severity of COVID-19 (33). Notably, an increasing number of researchers advocated for PCT testing in patients with cardiovascular diseases, including those with shortness of breath, possible heart failure, suspected endocarditis, and acute coronary syndrome (34). Our results suggested that elevated PCT levels in early-stage Omicron infection patients were associated with an abnormal increased serum markers for the cardiovascular system most significantly. Another noteworthy observation was that patients with abnormal indicators required a longer time for viral RNA clearance, resulting in an overall extension of the infection duration, patients with clearance times exceeding 14 days exhibited significantly elevated levels of BNP and DD. This implied that patients with abnormal increased serum markers for the cardiovascular system might experience a longer period of Omicron infection.

To the best of our knowledge, this study is the first to analyze five serum markers for cardiovascular system injury (CK-MB, cTnT, Myo, BNP, and DD) in the early stages of Omicron infection. Nevertheless, there are several limitations to this study. Firstly, it is a retrospective study conducted in a single medical center. Secondly, due to the challenges associated with emergency measurements, data such as the presence of underlying diseases were self-reported, potentially introducing recall biases. Thirdly, the study is limited by the availability of relevant research, and our focus is solely on specific markers, with all serum samples collected in the early stages of hospitalization. Lastly, there is no follow-up of patients about cardiac testing or echocardiogram to assess cardiovascular function. Future studies should conduct a comprehensive analysis of additional test results, and long-term follow-up studies on Omicron patients to explore the specific impacts on the cardiovascular system.

## Conclusion

Our findings suggested that even among asymptomatic or mild patients with Omicron infection, there might be an occurrence of abnormally elevated levels of cardiovascular indicators, signifying a certain degree of risk for subclinical cardiovascular damage. The abnormal cardiovascular markers were strongly associated with increased PCT levels. Female gender and age over 35 years old were also identified as contributing risk factors. We believed that this knowledge will play a crucial role in developing early intervention strategies to effectively address these risks.

## Data Availability

The raw data supporting the conclusions of this article will be made available by the authors, without undue reservation.

## References

[1] Ruan Q Yang K Wang W Jiang L Song J. Clinical predictors of mortality due to COVID-19 based on an analysis of data of 150 patients from Wuhan, China. Intensive Care Med. (2020) 46:846–48. 10.1007/s00134-020-05991-x 32125452 PMC7080116

[2] Chopra V Flanders SA O'Malley M Malani AN Prescott HC. Sixty-day outcomes among patients hospitalized with COVID-19. Ann Intern Med. (2021) 174:576–78. 10.7326/M20-5661 33175566 PMC7707210

[3] Sandoval Y Januzzi JJ Jaffe AS. Cardiac troponin for assessment of myocardial injury in COVID-19: JACC review topic of the week. J Am Coll Cardiol. (2020) 76:1244–58. 10.1016/j.jacc.2020.06.068 32652195 PMC7833921

[4] Henning RJ. Cardiovascular complications of COVID-19 severe acute respiratory syndrome. Am J Cardiovasc Dis. (2022) 12:170–91. 36147783 PMC9490160

[5] Xie Y Xu E Bowe B Al-Aly Z. Long-term cardiovascular outcomes of COVID-19. Nat Med. (2022) 28:583–90. 10.1038/s41591-022-01689-3 35132265 PMC8938267

[6] WHO Emerging Diseases Clinical Assessment and Response Network. WHO reference number: WHO/2019-nCoV/therapeutics/2022.2.March 3, 1–109. (2022).

[7] Puntmann VO Carerj ML Wieters I Fahim M Arendt C Hoffmann J Outcomes of cardiovascular magnetic resonance imaging in patients recently recovered from coronavirus disease 2019 (COVID-19). JAMA Cardiol. (2020) 5:1265–73. 10.1001/jamacardio.2020.3557 32730619 PMC7385689

[8] Rajpal S Tong MS Borchers J Zareba KM Obarski TP Simonetti OP Cardiovascular magnetic resonance findings in competitive athletes recovering from COVID-19 infection. JAMA Cardiol. (2021) 6:116–18. 10.1001/jamacardio.2020.4916 32915194 PMC7489396

[9] Maslo C Friedland R Toubkin M Laubscher A Akaloo T Kama B. Characteristics and outcomes of hospitalized patients in South Africa during the COVID-19 omicron wave compared with previous waves. JAMA. (2022) 327:583–84. 10.1001/jama.2021.24868 34967859 PMC8719272

[10] Dyer O. COVID-19: omicron is causing more infections but fewer hospital admissions than delta, South African data show. Br Med J. (2021) 375:n3104. 10.1136/bmj.n3104 34916213

[11] Garrett N Tapley A Andriesen J Seocharan I Fisher LH Bunts L High asymptomatic carriage with the omicron variant in South Africa. Clin Infect Dis. (2022) 75:e289–92. 10.1093/cid/ciac237 35353885 PMC9383623

[12] Wayne CM Singh N. Clinical implications of B-type natriuretic peptide and N-terminal pro–B-type natriuretic peptide in the care of the vascular surgery patient. Semin Vasc Surg. (2014) 27:143–47. 10.1053/j.semvascsurg.2015.01.004 26073822

[13] Cohen AT Spiro TE Spyropoulos AC Desanctis YH Homering M Buller HR D-dimer as a predictor of venous thromboembolism in acutely ill, hospitalized patients: a subanalysis of the randomized controlled MAGELLAN trial. J Thromb Haemost. (2014) 12:479–87. 10.1111/jth.12515 24460645

[14] Zhou F Yu T Du R Fan G Liu Y Liu Z Clinical course and risk factors for mortality of adult inpatients with COVID-19 in Wuhan, China: a retrospective cohort study. Lancet. (2020) 395:1054–62. 10.1016/S0140-6736(20)30566-3 32171076 PMC7270627

[15] Gyongyosi M Alcaide P Asselbergs FW Brundel B Camici GG Martins P Long COVID and the cardiovascular system-elucidating causes and cellular mechanisms in order to develop targeted diagnostic and therapeutic strategies: a joint scientific statement of the ESC working groups on cellular biology of the heart and myocardial and pericardial diseases. Cardiovasc Res. (2023) 119:336–56. 10.1093/cvr/cvac115 35875883 PMC9384470

[16] Fiedler L Motloch LJ Jirak P Gumerov R Davtyan P Gareeva D Investigation of hs-TnI and sST-2 as potential predictors of long-term cardiovascular risk in patients with survived hospitalization for COVID-19 pneumonia. Biomedicines. (2022) 10:1–15. 10.3390/biomedicines10112889 36359409 PMC9687975

[17] Motloch LJ Jirak P Gareeva D Davtyan P Gumerov R Lakman I Cardiovascular biomarkers for prediction of in-hospital and 1-year post-discharge mortality in patients with COVID-19 pneumonia. Front Med (Lausanne). (2022) 9:906665. 10.3389/fmed.2022.906665 35836945 PMC9273888

[18] Cuthbertson BH Amiri AR Croal BL Rajagopalan S Alozairi O Brittenden J Utility of B-type natriuretic peptide in predicting perioperative cardiac events in patients undergoing major non-cardiac surgery. Br J Anaesth. (2007) 99:170–76. 10.1093/bja/aem158 17573389

[19] Cohen AT Alikhan R Arcelus JI Bergmann JF Haas S Merli GJ Assessment of venous thromboembolism risk and the benefits of thromboprophylaxis in medical patients. Thromb Haemost. (2005) 94:750–59. 10.1160/TH05-06-0385 16270626

[20] Khalid M Awan S Jatoi NN Jatoi HN Yasmin F Ochani RK Cardiac manifestations of the coronavirus disease-19: a review of pathogenesis, clinical manifestations, diagnosis, and treatment. Pan Afr Med J. (2021) 39:173. 10.11604/pamj.2021.39.173.27802 34584599 PMC8449581

[21] Elshazli RM Toraih EA Elgaml A El-Mowafy M El-Mesery M Amin MN Diagnostic and prognostic value of hematological and immunological markers in COVID-19 infection: a meta-analysis of 6,320 patients. PLoS One. (2020) 15:e0238160. 10.1371/journal.pone.0238160 32822430 PMC7446892

[22] Craig JC Broxterman RM Wilcox SL Chen C Barstow TJ. Effect of adipose tissue thickness, muscle site, and sex on near-infrared spectroscopy derived total-[hemoglobin+myoglobin]. J Appl Physiol (1985). (2017) 123:1571–78. 10.1152/japplphysiol.00207.2017 28935822

[23] Cheng R Liu C Yang J Yang Y Chen R Ding X Sex differences in the incidence and risk factors of myocardial injury in COVID-19 patients: a retrospective cohort study. Front Physiol. (2021) 12:632123. 10.3389/fphys.2021.632123 33664674 PMC7920972

[24] Ambrosino I Barbagelata E Corbi G Ciarambino T Politi C Moretti AM. Gender differences in treatment of coronavirus disease-2019. Monaldi Arch Chest Dis. (2020) 90:646–56. 10.4081/monaldi.2020.1508 33305554

[25] Burgi JJ Rosslein M Nolte O Wick P Garcia BR Stranders S Mild COVID-19 induces early, quantifiable, persistent troponin I elevations in elder men. Front Cardiovasc Med. (2022) 9:1053790. 10.3389/fcvm.2022.1053790 36531721 PMC9751190

[26] Kochi AN Tagliari AP Forleo GB Fassini GM Tondo C. Cardiac and arrhythmic complications in patients with COVID-19. J Cardiovasc Electrophysiol. (2020) 31:1003–08. 10.1111/jce.14479 32270559 PMC7262150

[27] Hammarsten O Ljungqvist P Redfors B Wernbom M Widing H Lindahl B The ratio of cardiac troponin T to troponin I may indicate non-necrotic troponin release among COVID-19 patients. Clin Chim Acta. (2022) 527:33–7. 10.1016/j.cca.2021.12.030 34998858 PMC8744390

[28] Stein SR Ramelli SC Grazioli A Chung JY Singh M Yinda CK SARS-CoV-2 infection and persistence in the human body and brain at autopsy. Nature. (2022) 612:758–63. 10.1038/s41586-022-05542-y 36517603 PMC9749650

[29] Mizera L Borst O. COVID-19 and the incidence of acute myocardial injury. Hamostaseologie. (2021) 41:356–64. 10.1055/a-1554-6416 34695852

[30] Lala A Johnson KW Januzzi JL Russak AJ Paranjpe I Richter F Prevalence and impact of myocardial injury in patients hospitalized with COVID-19 infection. J Am Coll Cardiol. (2020) 76:533–46. 10.1016/j.jacc.2020.06.007 32517963 PMC7279721

[31] Qiu H Li J Li J Li H Xin Y. COVID-19 and acute cardiac injury: clinical manifestations, biomarkers, mechanisms, diagnosis, and treatment. Curr Cardiol Rep. (2023) 25:817–29. 10.1007/s11886-023-01902-w 37314650

[32] Marconato M Abela IA Hauser A Schwarzmuller M Katzensteiner R Braun DL Antibodies from convalescent plasma promote SARS-CoV-2 clearance in individuals with and without endogenous antibody response. J Clin Invest. (2022) 132:1–16. 10.1172/JCI158190 35482408 PMC9197521

[33] Hu R Han C Pei S Yin M Chen X. Procalcitonin levels in COVID-19 patients. Int J Antimicrob Agents. (2020) 56:106051. 10.1016/j.ijantimicag.2020.106051 32534186 PMC7286278

[34] Li J Cao T Wei Y Zhang N Zhou Z Wang Z A review of novel cardiac biomarkers in acute or chronic cardiovascular diseases: the role of soluble ST2 (sST2), lipoprotein-associated phospholipase A2 (lp-PLA2), myeloperoxidase (MPO), and procalcitonin (PCT). Dis Markers. (2021) 2021:6258865. 10.1155/2021/6258865 34422136 PMC8371622

